# Human amnion epithelial cells induce M2 macrophage polarisation partially via M-CSF secretion but independently of extracellular vesicles *in vitro*

**DOI:** 10.3389/fimmu.2026.1723968

**Published:** 2026-01-29

**Authors:** Louise Zeijlon, Snehil Budhwar, Robert Lindau, Stefan Bencina, Helen Kaipe, Maria C. Jenmalm, Roberto Gramignoli, Johanna Raffetseder

**Affiliations:** 1 Division of Inflammation and Infection, Department of Biomedical and Clinical Sciences, Linköping University, Linköping, Sweden; 2 Department of Laboratory Medicine, Division of Pathology, Karolinska Institutet, Solna, Sweden; 3 Department of Laboratory Medicine, Karolinska Institutet, Stockholm, Sweden; 4 Clinical Immunology and Transfusion Medicine, Karolinska University Hospital, Stockholm, Sweden; 5 Unità Operativa Semplice Dipartimentale (UOSD) Cell Therapy Lab, Istituto di Ricovero e Cura a Carattere Scientifico (IRCCS) Giannina Gaslini, Genova, Italy

**Keywords:** amnion epithelial cells, extracellular vesicles, human macrophage, immune regulation, macrophage polarisation, macrophage colony-stimulating factor, pregnancy

## Abstract

Pregnancy requires major immunomodulatory changes, both systemically and locally, as the maternal immune system needs to be modulated to tolerate the semi-allogeneic foetus. Decidual macrophages and stromal cells, but also foetal tissues are involved in this immune tolerance, for example by inducing M2 macrophages and regulatory T cells. However, it is so far unknown whether foetal membrane cells such as amnion epithelial cells (AECs) can influence human macrophage polarisation. In this study, a human *in vitro* macrophage assay was employed to demonstrate that conditioned medium (CM) from AECs derived from term placentas induces M2 macrophage polarisation, and to compare AEC culture conditions aiming for efficient M2 polarisation. Macrophage colony-stimulating factor (M-CSF), a well-known M2-inducing cytokine, was found to be secreted by AECs, and M-CSF was partly responsible for the observed M2-polarising effect of AECs. In addition, the M2-polarising effect remained after removal of extracellular vesicles (EVs) from AEC-CM, suggesting the involvement of soluble but not of EV-associated mediators. Taken together, this study shows that AECs may contribute to the induction of the vital immunotolerant environment at the foetal-maternal interface. Based on their immunomodulatory effects observed here and in *in vivo* studies, AECs could be harnessed as cytotherapeutics for inflammatory disorders.

## Introduction

Pregnancy represents an immunological paradox since the foetus contains both maternal and paternal antigens (1, 2). For a successful pregnancy, it is therefore vital that both the maternal and the foetal immune systems are modulated to create tolerance, while at the same time sustaining defence against infections (1, 3). The placenta has a central role in regulating the immune environment at the foetal-maternal interface (1, 4), which is composed of both maternal tissues, *i.e.*, the uterine endometrium named “decidua” during pregnancy, as well as the foetally derived trophoblast and membranes (1). The innermost layer of the amniotic membrane is composed of amnion epithelial cells (AECs), which can be isolated after delivery (5). AECs differentiate early during pregnancy from the epiblast and retain a significant level of stem cell potency (6), highlighting their strong potential for use in the treatment of various diseases (7, 8). AECs have been reported to have immunoregulatory properties, and these effects can be mediated both via secretion of soluble proteins as well as via extracellular vesicles (EVs) (9).

EVs are nanosized membrane-enclosed particles that can be released by all cells and are involved in numerous biological processes (10). Alike soluble mediators, EVs can influence cells by delivering immunomodulatory signals such as cytokines or other proteins (10). Regarding the effects of human AECs on immune cells, both cell-mediated, EV-mediated, and EV-independent effects have been reported (9, 11–13). The immunomodulatory effects of AECs include the inhibition of both proliferation and activation of human T cells, B cells, and NK cells, as well as enhancement of regulatory T and B cells (12, 13). However, studies on human macrophages are so far lacking.

Macrophages play important roles in immune regulation and homeostasis. Based on the simplified concept of M1 and M2 macrophages, the M1 phenotype is considered a proinflammatory phenotype, being involved in the response against infections and tumours, whereas M2-like macrophages are considered anti-inflammatory and tissue-protective due to their involvement in immune regulation and tissue homeostasis (14). At the foetal-maternal interface, they have important roles in tissue remodelling and display a phenotype associated with anti-inflammatory and immunoregulatory functions (14–16). This applies both for macrophages in the decidua (14–16) as well as for Hofbauer cells, which are tissue-resident foetal macrophages found within the chorionic villi (15, 17). Decidual macrophages and Hofbauer cells share not only immune regulatory properties, but also express established M2 markers like the scavenger receptor CD163 and the pattern recognition receptor CD209 (15, 16). Regarding the effects of AECs on macrophages, there is evidence that AECs can affect macrophage polarisation and function in *ex vivo* and animal models (11, 18–23), but to the best of our knowledge, it is so far unknown whether human AECs and their secreted mediators can modulate human macrophages, and by what molecular mechanisms. Assessment of AEC-mediated effects on human macrophages is warranted to understand the role of these perinatal multipotent cells in local immune regulation during pregnancy, and to expand on their utility in the treatment of diseases, in particular inflammatory conditions (7, 8).

Hypothesising that AECs contribute to tolerance at the foetal-maternal interface via M2 macrophage polarisation, this study aimed to investigate if human AECs can polarise human macrophages into an M2-like phenotype in the absence of direct cell-to-cell interactions, and if so, by which mechanisms. We report that AEC-conditioned medium (AEC-CM) can inhibit M1 and promote M2 polarisation of human monocytes. Protein profiling of AEC-CM revealed that M-CSF, a well-known M2-inducing agent (16, 24), was secreted by AECs, and M-CSF was partly responsible for the M2-polarising effect of AECs in the macrophage polarisation assay. In addition, the M2-polarising effect remained after removal of EVs from AEC-CM, suggesting the involvement of soluble instead of EV-associated mediators. Collectively, our findings support a role for AECs in local immune regulation during pregnancy.

## Material and methods

### Ethics approval statement

Collection of human materials for this study was approved by the local ethics committees in Linköping (M39/08) for collection of blood samples from healthy volunteers for monocyte isolation, and at Karolinska Institutet (nr 2015/419-31/4) for collection of human placentas and AEC isolation. All participants gave their written informed consent.

### Isolation and cultivation of human AECs

Full-term human placentas were collected after uncomplicated singlet pregnancies and uncomplicated planned caesarean procedures from healthy women (n=22; median age 35.5, age range 18-45) at a gestational age of 39 weeks (median, range 37–42 weeks), at Karolinska Hospital (Stockholm, Sweden) after signed consent. Five women were primipara; the others were multipara with 1–4 previous births. Of the delivered babies, 13 were male and 9 female.

Isolation of human AECs was performed as previously described (5) within 100 min after delivery. Briefly, the amnion membrane was removed from the inner placental surface and washed to remove maternal blood. Epithelial cells were released from the amnion membrane by exposure to TrypLE Express (Dundee, UK) digestion and immediately seeded on rat collagen-treated culture vessels. Cells were maintained in low glucose Dulbecco’s Modified Eagle Medium (Thermo Fisher, Waltham, MA, USA) supplemented with non-essential amino acid (Thermo Fisher) and 5% human platelet lysate (Cook Regentec, Bloomington, IN, USA). Cell viability post-isolation was 93% (median), as assessed by Trypan Blue exclusion method, and cell identity was proven as previously described by static surface antigens (5).

### Collection of conditioned media from AECs

Of the freshly isolated, viable AECs, 120 000/cm^2^ were seeded in collagen pre-treated culture vessels and maintained in culture for 3 or 7 days under hypoxic (2% O_2_) or normoxic conditions. The culture medium was changed after 4 days. Culture medium at days 3 and 7 were collected and centrifuged at 1 000g for 10 min to remove apoptotic bodies and cellular debris .For three samples, conditioned medium was collected after 2 days of culture. These samples were analyzed together with samples cultured for three days, as specified in the figure legends. The pellet was discarded and supernatants collected and stored at -80°C for further analyses.

### Isolation of CD14^+^ cells

Heparinised whole blood was collected from healthy non-pregnant women (18 to 45 years of age), and peripheral blood mononuclear cells (PBMC) were isolated using a Lymphoprep (Dundee, UK) gradient according to a previously published protocol (25), featuring dilution of the blood with Hank’s Balanced Salt Solution (Gibco/Thermo Fisher), centrifugation at 400g for 30 min and washing of cells in Hank’s Balanced Salt Solution. Subsequently, CD14^+^ cells were isolated using positive magnetic bead cell sorting with MS columns (Miltenyi Biotec, Bergisch Gladbach, Germany) according to the manufacturer’s protocol.

### Macrophage polarisation assay

Macrophages were generated from CD14^+^ blood monocytes as previously described (4, 16, 26–28) by culturing 100 000 CD14^+^ cells/well in triplicates for 6 days in a 96-well plate at 37°C in Roswell Park Memorial Institute 1640 (RPMI) medium (Gibco/Thermo Fisher) supplemented with 10% FCS (GE Healthcare, Chicago, IL, USA) and 1% penicillin-streptomycin/L-glutamine (100 U/ml penicillin, 100 µg/ml streptomycin and 292 µg/ml L-glutamine, Gibco/Thermo Fisher). Cells were treated with 5 ng/ml GM-CSF (PeproTech, Cranbury, NJ, USA) to promote differentiation into an M1-like macrophage phenotype (16, 27), with or without addition of different concentrations of AEC-CM, or the EV-enriched and EV-depleted fractions of AEC-CM. As a control for M2 induction, M-CSF-treated cells were included in all experiments (50 ng/ml M-CSF, PeproTech) (16, 27). Cells were incubated at 37 °C in 5% CO_2_ in humidified atmosphere, and medium was replaced after 3 days. Flow cytometry was performed after 6 days of culture.

### Blocking experiments

M-CSF was neutralised in AEC-CM by the addition of 1 µg/ml anti-human M-CSF antibody (clone #26730, catalogue number: MAB216-500, R&D Systems, Minneapolis, MN, USA). The M-CSF antibody or its corresponding isotype control antibody (clone #20102, catalogue number: MAB003, R&D Systems) was added to RPMI medium containing AEC-CM, 5 ng/ml GM-CSF, 10% FCS and 1% penicillin-streptomycin/L-glutamine, and incubated in room temperature for 1 hour prior to the addition of CD14^+^ cells to allow for binding of the antibody to M-CSF. This procedure was repeated during medium change after 3 days of culture. To reduce the effect of the differential M2-inducing capacities seen between the individual AEC-CM samples, 12.5 or 25% AEC-CM was used.

Recombinant human VEGFR1/Flt-1 Fc chimera protein (catalogue number: 321-FL-050, R&D Systems) was used to neutralise VEGF at a concentration of 1.5 or 4.5 µg/ml. Concentrations were chosen based on literature and prior screening. Briefly, the VEGFR1/Flt-1 was added to RPMI medium containing 25% AEC-CM supplemented with 10% FCS and 1% penicillin-streptomycin/L-glutamine and incubated at 37°C for 30 min prior to adding to the cells.

### Relative quantification of AEC-secreted proteins by proximity extension assay

One μl of AEC-CM were analysed by the SciLifeLab Affinity Proteomics at Uppsala University, Sweden for 92 inflammation-associated proteins using the Olink inflammation panel (https://olink.com/products-services/target/inflammation/) with proximity extension assay (PEA) as previously described (27). Data are expressed as normalised protein expression (NPX) on a log2 scale.

### EV isolation

For isolation of EVs, one ml AEC-CM was subjected to three pre-centrifugation steps (300g, 2 000g and 10 000g for 5, 20 and 60 min, respectively, all at 4°C) to remove apoptotic bodies and cellular debris. The supernatant was then placed into 1 ml ultracentrifuge tubes (PC TK WALL, Beckman Coulter, Brea, CA, USA) and ultracentrifuged for 70 min at 100 000g at 4°C (Optima MAX-XP, Beckman Coulter). The supernatant was then removed and saved as the EV-depleted fraction, and the pellet was resuspended and washed in sterile-filtered PBS and centrifuged again at 100 000g for 70 min at 4°C. The supernatant was removed and the EV pellet was dissolved overnight at 4°C in RPMI medium containing 1% penicillin-streptomycin/L-glutamine and 1% amphotericin B (250 µg/ml, Gibco/Thermo Fisher) to obtain the EV-enriched fraction. For this, we used the same volume as for the initial AEC-CM sample (1 ml), to allow for comparison of the EV-depleted and EV-enriched fraction.

### EV characterisation

Western blotting was performed on both the EV-enriched and the EV-depleted fractions. Briefly, lysate from both fractions were examined on 4-12%, Bis Tris, mini protein gels (Invitrogen/Thermo Fisher). The transferred PVDF membrane was developed using Pierce™ ECL Western Blotting Substrate (Thermo Fischer) and imaged using a ChemiDoc Imager (BioRad, Hercules, CA, USA). The primary antibodies used were rabbit monoclonal anti-human flotillin-1 (1:1000 dilution, D2V7J, CST) for the positive EV marker, flotillin-1 and rabbit anti-human calnexin (1:1000 dilution, clone EPR3632, Abcam) for the negative EV marker calnexin. The secondary antibody employed was HRP-tagged goat anti-rabbit IgG (1:2000 dilution, Agilent Dako).

The expression of surface proteins on EVs was analysed in the EV-enriched and EV-depleted fractions using the human MACSPlex Exosome kit (Miltenyi Biotec) according to the manufacturer’s instructions. In brief, samples with an adjusted protein amount of 5-10 µg were diluted with MACSPlex buffer and incubated overnight with capture beads containing 39 antibody-coated bead subsets. The EV bead complexes were then washed using MACSPlex buffer and subsequently incubated with APC-conjugated detection antibody mixture (CD81, CD9, and CD63) before acquisition on a Gallios flow cytometer (Beckman Coulter). Data were analysed in Kaluza (Beckman-Coulter) software version 2.1.

The morphology of the isolated EVs was examined through transmission electron microscopy (TEM). Briefly, 5 μl EV suspension was fixed in 2% paraformaldehyde. The fixed sample was applied on formvar/carbon-coated copper grids (size 300 mesh, Ted Pella, Redding, CA, USA) for 10 min. The grids were rinsed twice for 1 min each with 1X PBS, followed by blotting and staining with 2% uranyl acetate for 30 seconds. Excess uranyl acetate was removed by blotting on filter paper. The grids were air-dried for 5–10 min and then visualised using an 80kV transmission electron microscope (JEOL JEM-1400Flash, JEOL Ltd., Tokyo, Japan).

The size of EVs was measured by nanoparticle tracking technology using the NanoSight NS300 instrument (Malvern, UK) as per the manufacturer’s instruction. Briefly, the samples were diluted in sterile-filtered PBS to obtain 30–80 particles per frame and gently vortexed before being injected (25°C) with a syringe load with constant flow injection. Five videos of 60 s were captured with camera level 16 and detection threshold 5. Data was analysed using the NanoSight NTA software 3.4 (Malvern).

### Flow cytometry

After 6 days of incubation in AEC-CM from different culture conditions, and polarisation and blocking factors, the expression of macrophage markers CD163, CD14, CD209 and HLA-DR was analysed using flow cytometry. Cells were washed once with sterile PBS (Gibco/Thermo Fisher) before incubation with TrypLE for 15 min at 37°C for detachment. 100 µl of RPMI medium supplemented with 10% FCS and 1% penicillin-streptomycin/L-glutamine was used to stop the TrypLE reaction. Thereafter, the cells were centrifuged at 500g for 5 min, the supernatant was discarded, and the cells were resuspended in 200 µl PBS supplemented with 1% FCS and 0.2% Aqua Live/Dead Cell Stain (Invitrogen). The cells were then stained with the fluorescent antibodies or the appropriate isotype control antibodies (**Table 1**) and incubated for 30 min at 4°C, before they were washed once more with 2 ml PBS supplemented with 1% FCS and centrifuged at 500g for 5 min. Cells were resuspended in 100 µl PBS supplemented with 1% FCS.

**Table 1:** Antibodies used for flow cytometric analysis of macrophage phenotypes.

Fluorochrome	Analysis antibody			Isotype control antibody		
	Marker	Clone	Manufacturer	Isotype	Clone	Manufacturer
PE	CD163	GHI/61	BD Pharmingen	IgG1	OX40	BD
PerCp-Cy5.5	CD209	DCN46	BD Pharmingen	IgG2b	27-35	BD Pharmingen
PC7	CD14	RMO52	Beckman Coulter	IgG2a	7T4-1F5	Beckman Coulter
FITC	HLA-DR	L243	BD Biosciences	IgG2b	27-35	BD Pharmingen

**Figure 1:**
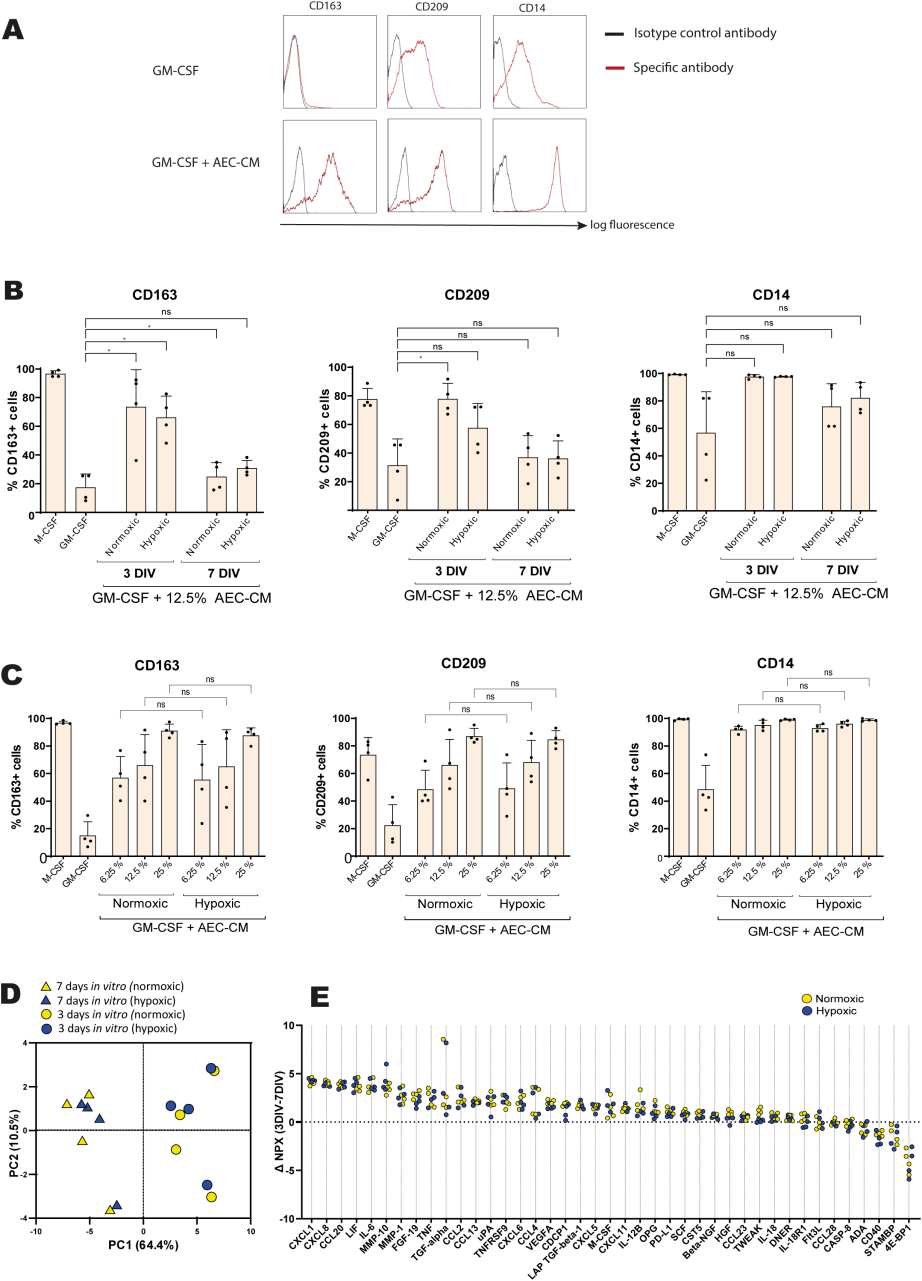
M2-inducing properties and protein profiling of human amnion epithelial cell-conditioned medium (AEC-CM) from different culture conditions. **(A-C)** CD14^+^ cells from human PBMCs were cultured in presence of the M1-associated growth factor GM-CSF and in presence and absence of AEC-CM, followed by flow cytometric analysis of macrophage markers after 6 days of culture. Cells treated with M-CSF served as a positive control for M2 induction. **(A)** Representative flow cytometry histograms showing that 12.5% AEC-CM induce the regulatory markers CD163, CD209 and CD14. **(B)** In a first screening, 12.5% conditioned media from AECs from four placentas cultured in normoxic or hypoxic conditions for either 3 (3DIV) or 7 (7DIV) days in vitro were added to monocytes treated with GM-CSF. Statistical differences between AEC-CM-treated samples and samples treated with only GM-CSF were tested using repeated measures one-way ANOVA with Dunnett’s multiple comparison test. **(C)** 6.25, 12.5, and 25% AEC-CM from four placentas cultured in normoxic or hypoxic conditions for 3 days were added to monocytes treated with GM-CSF. Statistical differences between samples treated with AEC-CM from normoxic and hypoxic culture were tested using repeated measures one-way ANOVA with Sidak’s multiple comparison test. In **(B, C)**, bars and error bars represent means and standard deviations. ns, not significant, *p<0.05. **(D)** Principal component analysis (PCA) plot on protein levels obtained from analysis of 92 immune- and inflammation-associated proteins in paired AEC-CM samples. **(E)** Delta-NPX-values for individual proteins were calculated by subtracting 7DIV from 3DIV values for paired samples for normoxic (yellow) and hypoxic (blue) conditions. Delta-NPX-values above 0 indicate higher protein levels in the 3DIV samples, and delta-NPX below 0 indicates higher protein levels in the 7DIV samples. For visualisation, proteins are ranked based on median delta-NPX values. Each dot represents one sample from normoxic or hypoxic culture. In **(D, E)**, all proteins with less than 50% missing values (n=41) for all samples are shown.

Flow cytometric data were acquired on a FACS Canto II (BD Biosciences) using FACSDiva Software version 8. The gating was performed on Kaluza software version 2.1. After selection of the macrophage population based on FSC and SSC, singlet and alive macrophages were selected based on FSC-A and FSC-H and the absence of staining with the Aqua Live/Dead Cell Stain, respectively (**Supplementary Figure S1**). Among these, the percentage of cells positive for each marker was analysed by gating based on the corresponding isotype control sample (**Figure 1A**). For relative expression of markers, the median fluorescence intensity (MFI) of each marker was divided with the MFI of its corresponding isotype control sample to obtain the MFI ratio.

### Statistics

To test the difference in M2-inducing capacity between different culture conditions of AEC-CM, differences between AEC-CM and GM-CSF-treated samples, the effect of VEGFR-blocking, and whether the EV-enriched and -depleted fractions differed from the original AEC-CM samples, repeated measures one-way ANOVA with Dunnett’s multiple comparison test was performed. To test differences between normoxic and hypoxic AEC-CM, repeated measures one-way ANOVA with Sidak multiple comparison *post hoc* test was carried out. To test the effect of M-CSF blocking, a paired t-test was performed. A p-value of ≤0.05 was considered statistically significant. GraphPad Prism version 9 (La Jolla, CA, USA) was used to perform all statistical analyses. Results from non-parametric testing did not reveal any major differences from parametric testing, thus we report results from the parametric tests.

## Results

### The M2 macrophage-inducing effects and the proteomic profile of AEC-derived conditioned medium

Primary human AECs were isolated from placentas collected at term after caesarean sections. All AEC samples exhibited homogeneity in composition, with epithelial markers above 99% (data not shown), in line with what was described previously (5). To evaluate the M2-inducing capacity of factors secreted by human AECs, we used a previously established *in vitro* macrophage polarisation model (4, 16, 26–28). CD14^+^ monocytes were isolated from the blood of healthy non-pregnant women, polarised towards an M1-like phenotype by GM-CSF, and analysed by flow cytometry. In parallel, AEC-CM obtained from different cultivation conditions was added to investigate if AEC-CM could override GM-CSF-induced M1 polarisation.

As a first screening, AEC-CM was obtained from cultures from different periods of time (3 and 7 days) and in normoxic or hypoxic conditions (the latter to mimic physiological conditions in the uterus), which might affect AEC-secreted factors. 12.5% AEC-CM from 3 days of *in vitro* (3DIV) culture in both normoxic and hypoxic conditions were able to override GM-CSF-induced M1-polarisation as seen by the significant increase in the percentage of macrophages expressing the M2-associated marker CD163 (**Supplementary Figure S1**; **Figures 1A, B**). A similar result was noted for the other major M2 marker CD209 (**Figures 1A, B**), with statistical significance obtained for AEC-CM generated in normoxic (*P*=0.04) but not in hypoxic conditions (*P*=0.33). As for other phenotypic markers, no significant differences were noted for the M2 marker CD14 or the M1-associated activation marker HLA-DR (**Figures 1A, B**; **Supplementary Figure S2A**). In contrast to CM from AECs cultured for 3DIV, AEC-CM collected after 7 days *in vitro* (7DIV) was unable to induce M2 polarisation, the only exception being a numerically small but statistically significant increase in CD163-expressing cells in normoxic conditions (**Figure 1B**). As for cellular expression of the markers – expressed as median fluorescence intensity (MFI) – AEC-CM cultured for 3DIV was able to increase the expression of CD14 as compared with the GM-CSF-induced phenotype. Otherwise, there were no significant differences in MFI for CD163, CD209 or HLA-DR (**Supplementary Figure S2B**). Given the less prominent M2-polarising effect of AEC-CM after 7DIV culture, the choice was made to proceed with AEC-CM from cells cultured for 3 days.

Next, the influence of normoxic and hypoxic conditions was evaluated in depth in the 3DIV samples by testing additional concentrations of AEC-CM (besides 12.5 also 6.25 and 25%). Samples from both normoxic and hypoxic AEC culture induced a dose-dependent increase in M2 polarisation (**Figure 1C**; **Supplementary Figures S2C, D**). However, no significant difference in neither the proportion nor the MFI of M2-like cells between normoxic *versus* hypoxic conditions was found (**Figure 1C**; **Supplementary Figures S2C, D**).

To verify the functional differences of the different culture conditions at a secretory level, protein profiling of AEC-CM was performed using a targeted proximity extension assay (PEA) including 92 immune and inflammation-associated proteins (**Supplementary Table 1**). PEA combines high sensitivity with high specificity, and data are presented on a log2 scale as normalised protein expression (NPX), allowing for relative comparisons between samples. In all subsequent analyses, proteins with less than 50% missing data (n=41) were included. A principal component analysis (PCA, **Figure 1D**) confirmed the influence of length of culture with a clear difference in the protein abundance and profile for 3 days compared to 7 days of *in vitro* culture, while normoxic *versus* hypoxic culture conditions did not reveal any apparent influence. Thus, protein profiles correlated well with the findings from the M2 macrophage polarisation assay. To investigate which proteins differed most between the 3DIV and 7DIV samples, delta-NPX values were obtained by subtraction of paired 7DIV from 3DIV NPX values. More than 80% of the proteins (34 out of 41) had a higher NPX value in 3DIV compared to 7DIV as indicated by a median delta-NPX value above zero (**Figure 1E** and **Supplementary Table 1**). The largest relative differences between 3DIV and 7DIV samples were observed for C-X-C motif ligand 1 (CXCL1), CXCL8 and C-C motif ligand 20 (CCL20), which were all higher in 3DIV samples, and for eukaryotic translation initiation factor 4E-binding protein 1 (4E-BP1), STAM-binding protein (STAMBP) and CD40, which were all higher in 7DIV samples. In contrast to length of culture, oxygen pressure did not influence the secreted protein profile (**Figure 1E**, statistical comparison not shown). Collectively, these screening data, despite the low number of samples, consistently indicated the importance of length of culturing, while no effects of hypoxic *versus* normoxic conditions were noted. Based on these observations, and since a progressive loss in AEC secretory capacity has been previously described during extended *in vitro* culture (29), subsequent analyses were limited to AEC-CM generated by 3 days of *in vitro* culture in normoxic conditions.

### AEC-CM induces M2 macrophages in a dose-dependent manner

**Figure 2:**
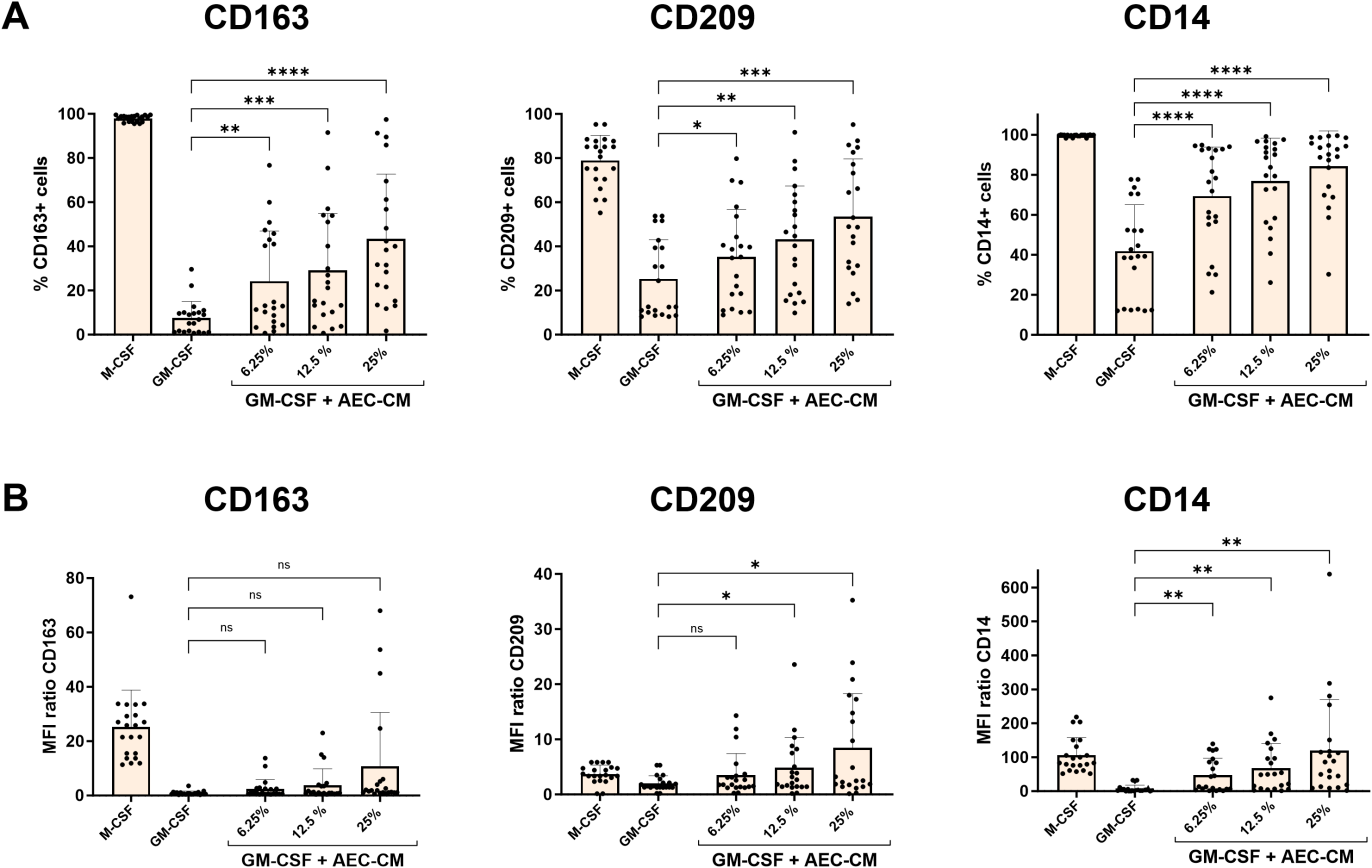
AEC-CM overrides the M1-polarising effect of GM-CSF and induces M2-macrophage polarisation in a dose-dependent manner. 6.25, 12.5 and 25% CM from 3 days of normoxic AEC culture were added to monocytes treated with GM-CSF and analysed by flow cytometry for **(A)** the percentage of cells positive for the macrophage markers CD163, CD209, and CD14, and **(B)** for their relative expression (MFI ratio) after 6 days of differentiation. Bars and error bars represent means and standard deviations. Statistical differences between AEC-CM-treated samples and samples treated with only GM-CSF were tested using repeated measures one-way ANOVA with Dunnett’s multiple comparison test. n.s. not significant, * p<0.05, ** p<0.01, ***p<0.001, ****p<0.0001. n=21, out of which two AEC-CM samples were from only 2 days of normoxic culture. MFI: median fluorescence intensity.

After establishing optimal AEC culture conditions for efficient M2 induction by AEC-CM, we aimed to verify the dose dependency of M2 induction in a larger set of AEC-CM samples (n=21), using the same *in vitro* macrophage polarisation assay. Monocytes cultured in the presence of GM-CSF and in increasing concentrations of AEC-CM from 3 days of normoxic *in vitro* culture (6.25%, 12.5%, and 25%) were polarised towards M2 macrophages shown by the increased percentage of cells positive for the M2-markers CD163, CD209 and CD14, as compared to monocytes stimulated with GM-CSF only (**Figure 2A**). The cellular expression of CD163 in terms of MFI did not increase significantly in comparison with macrophages polarised with GM-CSF alone, however, MFI of CD209 significantly increased when the cells were treated with 12.5% and 25% AEC-CM, and MFI of CD14 significantly increased compared to GM-CSF in a dose-dependent manner (**Figure 2B**). No differences were found in the percentage of HLA-DR positive cells between the different concentrations of AEC-CM (**Supplementary Figure S3A**), but the MFI of HLA-DR significantly decreased with 25% AEC-CM (**Supplementary Figure S3B**). To summarise, AEC-CM efficiently inhibits GM-CSF-induced M1 polarisation and induces M2 macrophages in a dose-dependent manner.

### M-CSF is partly responsible for the M2-polarising effect of amnion epithelial cells

**Figure 3:**
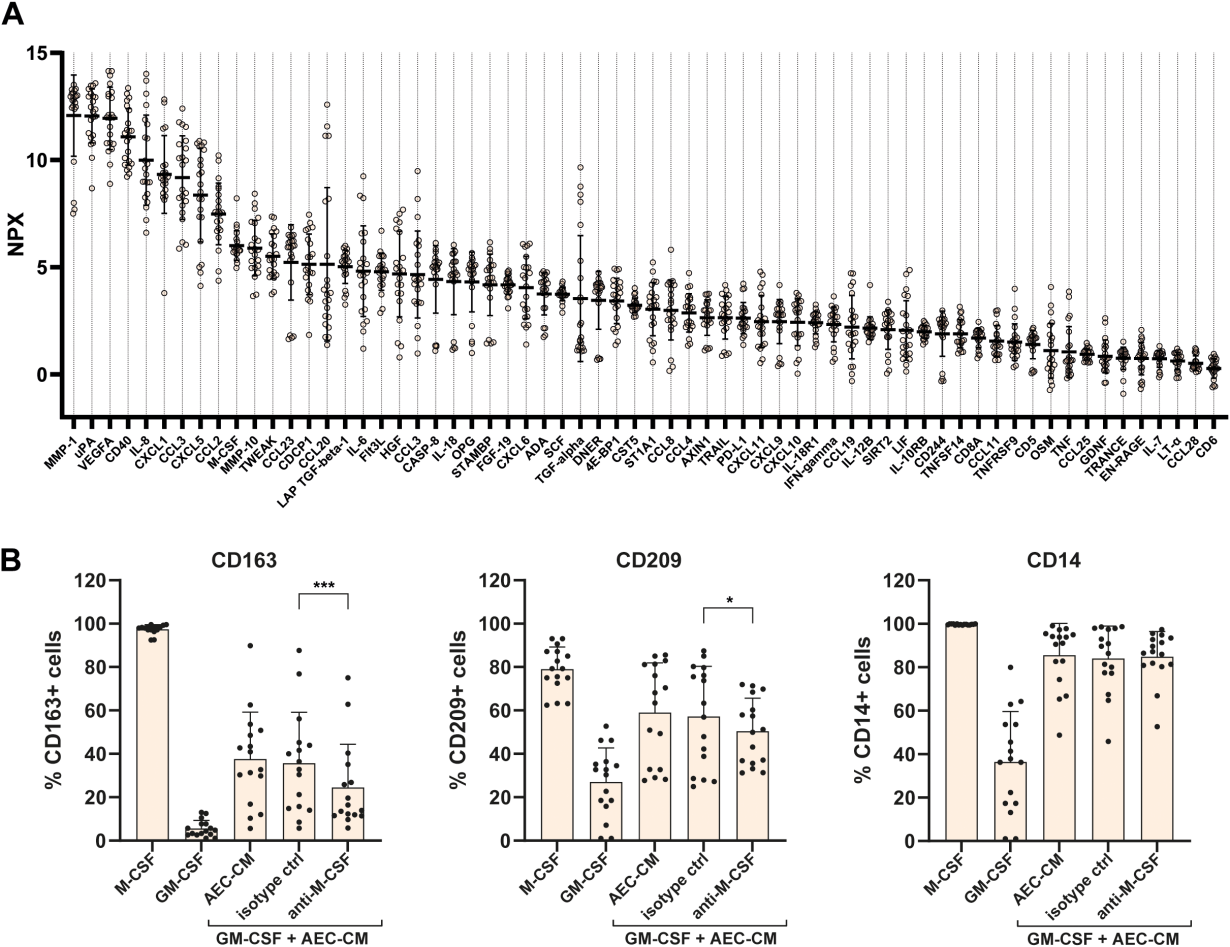
M-CSF is secreted by AECs and partly responsible for the M2-polarising effect of AEC-CM. **(A)** Protein profiling of 92 immune- and inflammation-associated proteins by proximity extension assay in AEC-CM cultured for 3 days in normoxic conditions. The mean NPX value and standard deviation of all proteins with less than 50% missing values are shown (n=59). Proteins were ranked based on descending mean values. Note that PEA quantifies protein levels in a relative manner; hence, NPX values cannot be compared between individual proteins. Each dot represents one sample. n=22, out of which three AEC-CM samples were from only 2 days of normoxic culture. **(B)** 12.5 (n=3) or 25% (n=13) of AEC-CM were added to monocytes treated with GM-CSF in the presence or absence of an anti-M-CSF antibody or an isotype control antibody (ctrl) and cultured for 6 days prior to flow cytometric analysis. To test for differences between cells treated with the M-CSF and the isotype control antibody, a paired t test was performed. * p<0.05, *** p<0.001. Bars and error bars represent mean and standard deviation. n=16, out of which three AEC-CM samples were from only 2 days of normoxic culture.

To further investigate the proteins potentially responsible for the M2-polarising effect of AEC-CM, PEA was performed on a larger set of AEC-CM samples obtained from 3 days of *in vitro* culture under normoxic conditions (n=22, **Figure 3A**; **Supplementary Table 2**). Out of the 92 proteins analysed in the PEA panel, 59 proteins were detectable in AEC-CM with less than 50% missing data. Among these AEC-secreted proteins, M-CSF and VEGF are known to be able to influence macrophage polarisation. M-CSF is a well-established M2 macrophage-inducing growth factor known for its role in M2 polarisation of decidual macrophages (16, 24). To determine if M-CSF is also responsible for the M2-inducing effect of AEC-CM, blocking of M-CSF in AEC-CM was performed by the addition of an M-CSF neutralising antibody. Blocking of M-CSF significantly decreased the proportion of cells expressing the M2 markers CD163 and CD209 compared to cells treated with an isotype control antibody, but did not change the proportion of cells expressing CD14 or HLA-DR (**Figure 3B**; **Supplementary Figure S4A**). As for cellular expression (MFI ratio), CD14 decreased (**Supplementary Figure S4B**), while CD163 and CD209 did not change significantly (**Supplementary Figure S4B**). The MFI ratio of HLA-DR increased compared with the isotype control antibody-treated sample (**Supplementary Figure S4B**). Thus, M-CSF is at least partly responsible for the M2-polarising effect of AEC-CM.

PEA also showed secretion of vascular endothelial growth factor (VEGF, **Figure 3A**; **Supplementary Table 2**), and since VEGF is able to induce M2 macrophages (30, 31), neutralisation of VEGF with a soluble VEGF receptor was performed. However, VEGF neutralisation in AEC-CM had no effect on M2 macrophage polarisation (**Supplementary Figure S5**).

### Extracellular vesicles from AEC-CM do not mediate M2 polarisation

**Figure 4:**
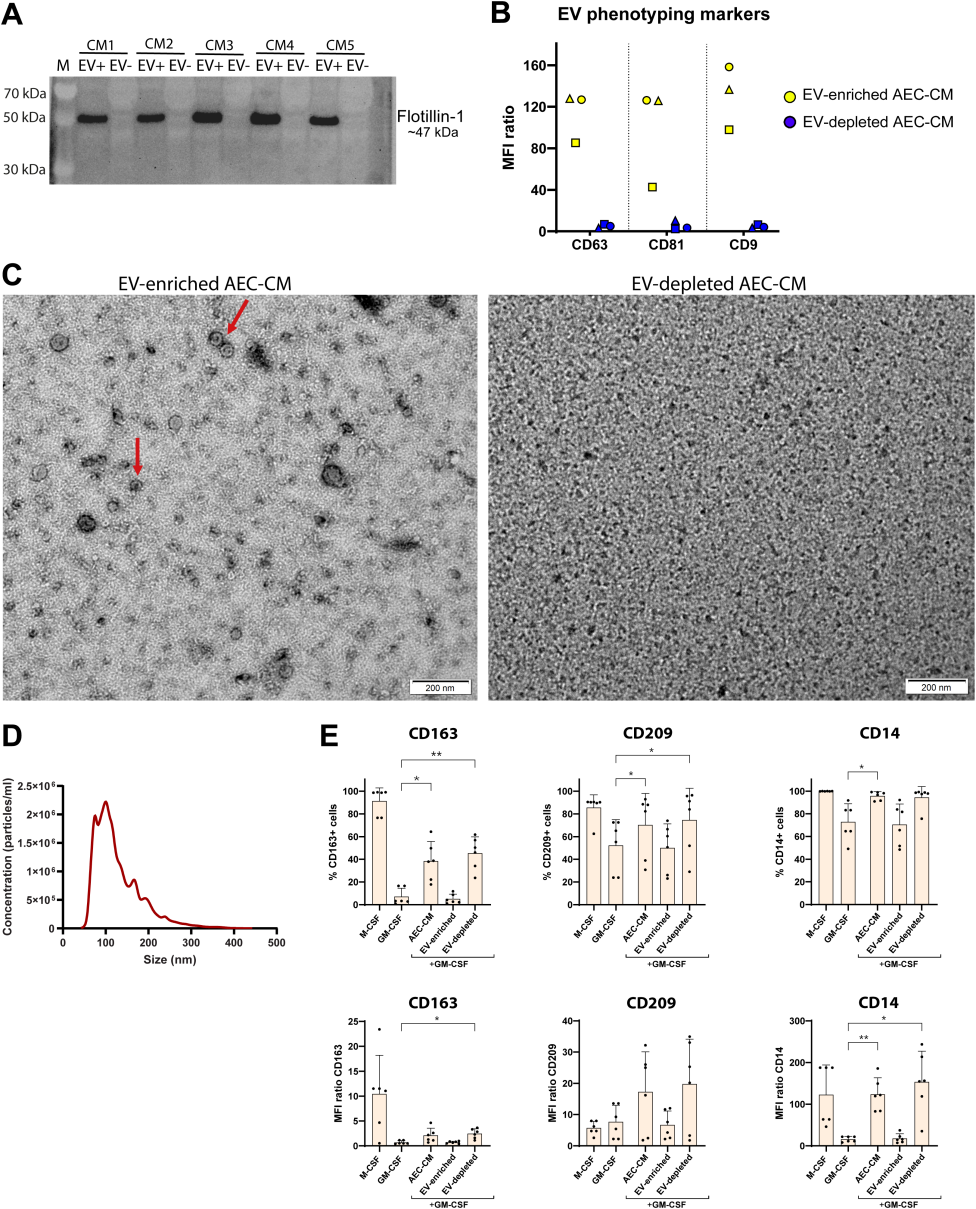
The extracellular vesicle (EV)-depleted fraction but not EVs from AEC-CM induce M2 polarisation. EVs were isolated by ultracentrifugation and EV-enriched and -depleted fractions were characterised based on current guidelines. **(A)** Western blot of the EV marker flotillin-1 in the EV-enriched (EV+) and EV-depleted (EV-) fraction of five randomly chosen AEC-CM samples from 3 days of normoxic culture. CM 1–5 indicate the different AEC-CM samples. **(B)** MFI ratio from flow cytometric analysis of EV phenotype markers (n=3). Each symbol represents one individual sample. **(C)** Transmission electron microscopy pictures of the EV-enriched and the EV-depleted fraction of one representative AEC-CM sample, showing EVs in the enriched fraction (arrows) but their absence in the depleted fraction. **(D)** Size distribution of the EV-enriched fraction of AEC-CM as quantified by nanoparticle tracking technology, presented as geometric mean from 5 samples. **(E)** The EV-enriched fraction and the EV-depleted fraction as well as the original AEC-CM sample (labelled “AEC-CM”) were added to monocytes at a concentration of 25% together with GM-CSF to investigate the M2 macrophage-inducing capacity n=6, out of which one AEC-CM sample was from only 2 days of normoxic culture. Top row: the percentage of positive cells for each marker. Bottom row: the MFI ratio for each marker. Statistical differences were tested using repeated measures one-way ANOVA with Dunnett’s multiple comparison test, * p<0.05, **p<0.01. Bars and error bars represent mean and standard deviation.

Since it has been proposed that AECs exert immune modulating effects through secretion of EVs (11, 32), we investigated if M2 polarisation of human macrophages was induced by EVs or by the secretion of soluble factors. EVs were therefore isolated by ultracentrifugation from a subset of the previously analysed AEC-CM samples, and EV isolation was confirmed according to the current guidelines (33) by Western blotting and flow cytometry of EV-associated markers, as well as by transmission electron microscopy (TEM). Western blotting of five randomly selected AEC-CM samples confirmed the presence of EVs in the EV-enriched fraction, demonstrated by presence of the EV marker flotillin-1, while flotillin-1 was absent from the EV-depleted fraction (**Figure 4A**; **Supplementary Figure S6**). Furthermore, the EV-enriched fraction was negative for calnexin, which is a chaperone protein present in the endoplasmic reticulum and thus a marker for cell contamination (**Supplementary Figure S6B**). Flow cytometry revealed that the EV-enriched fractions were positive for the EV surface markers CD63, CD81, CD9, CD29, CD142, CD41b, CD42p and SSEA4, while these markers were not detected in the EV-depleted fractions (n=3, **Figure 4B**; **Supplementary Figure S7**). TEM on the same three AEC-CM samples confirmed the presence of EVs in the EV-enriched fraction and absence of EVs in the EV-depleted fractions of AEC-CM (**Figure 4C**). Based on Nanosight nanoparticle tracking analysis, the EV concentration in the EV-enriched fraction was 1.62x10^12^ particles/ml (median of n=5, range 3.7x10^11^ – 3.6x10^12^). The size of isolated EVs was below 200 nm (**Figure 4D**; median of means of 5 samples; 129 nm, range 112–150 nm), which could indicate that they are exosomes (33). For EV-depleted fractions, no NTA data could be recorded due to too low particle counts. In summary, the characteristics of the EVs isolated in our study are in agreement with findings in other studies on human AEC-derived EVs (9, 13, 32, 34).

After confirming the presence or absence of EVs in the respective fractions, the M2-inducing effects of purified EVs isolated from AEC-CM were compared with the EV-depleted CM from the same sample using the same *in vitro* assay as before. Notably, the M2-inducing properties of AEC-CM were seen for the EV-depleted fraction of AEC-CM, as shown by the significantly increased proportion of the M2-markers CD163 and CD209 compared with the GM-CSF-treated control. In contrast, EVs isolated from AEC-CM were not able to induce M2-like macrophages, as shown by the similar proportions of CD163- and CD209-expressing cells compared to cells treated with GM-CSF only (**Figure 4D**). The same pattern was noted for the cellular expression (MFI ratio) of CD14 and CD163, but not for CD209. There were no significant differences in the proportions of CD14-expressing cells or in the proportion and MFI ratio of HLA-DR expressing cells (**Figure 4D** and **Supplementary Figure S8**). In summary, EVs from AECs did not mediate the M2-polarising effect; instead the M2-polarising effect can be attributed to other secreted factors in the CM.

## Discussion

Based on their immunomodulatory, anti-inflammatory and tissue repair properties, human AECs have been considered as stem cell-based therapy in models of inflammatory and auto-immune diseases (8, 9), and a few clinical trials have confirmed their safety and efficacy (8). Besides the cells *per se*, AEC-conditioned medium and AEC-derived EVs have been investigated as treatment option (35). So far, a complete picture of their immunomodulatory actions had been missing, and their effects on human macrophages have not been investigated.

AEC-CM from 3 days of culture effectively induced M2 polarisation, but this could not be achieved by AEC-CM from 7-day cultures. Pinpointing the reasons for this was beyond the scope of this study, but could be due to the generally lower production of most of the investigated proteins in AEC-CM from 7-day culture compared to 3-day culture. However, some proteins were present at higher levels in AEC-CM from 7-day culture, and these increased concentrations of 4E-BP1, STAMBP and CD40 could point to a role in M1 polarisation or in inhibition of M2 polarisation. Furthermore, AEC-CM from 3-day culture contained higher levels of M-CSF, a known M2 inducer (16, 24), but also higher levels of several inflammatory proteins such as CXCL1 and CXCL8, the latter seemingly contradicting M2 polarisation. Since we performed targeted proteomic analysis limited to 92 proteins, we did not get a complete picture of the proteomic profiles of AECs, and the observed overriding of M1 polarisation is likely due to a complex interaction of mediators in AEC-CM.

While both functional and proteomic analyses revealed clear differences between different time periods for generation of CM, we did not see any differences between CM that was obtained from normoxic and hypoxic culture. This contrasts with a study where AECs cultured in hypoxic conditions expressed elevated levels of IL-6 and CCL2 compared to cells cultured in normoxic conditions (36). However, that study analysed AEC-CM from only one single donor, and culture conditions differed significantly from our setup. Hence, the secretome of AECs seems to be highly dependent on AEC culture conditions, which warrants careful evaluation of culture conditions in future studies on AECs.

AEC-CM cultured for 3 days under normoxia efficiently induced M2-macrophage polarisation; thus, the choice was made to use a larger number of AEC-CM samples from these culture conditions to verify the ability of AEC-CM to inhibit M1 and to induce M2 polarisation. M2 polarisation by human AEC-CM has previously been shown in murine models *in vitro* (11, 23), but to the best of our knowledge this is the first study demonstrating that AEC-CM also induces human M2 macrophages. Given that proteomic analysis of AEC-CM from different culture conditions did not yield conclusive information regarding the potential M2-inducing proteins at play, we investigated whether M-CSF could be involved. Blocking of M-CSF revealed its partial involvement in inhibition of GM-CSF-induced M1-induction in our setup, but it failed to account for the whole effect. Targeted protein profiling indicated high concentrations of VEGF in AEC-CM from 3-day normoxic culture. Given the role of VEGF in promoting M2 polarisation (30, 31), we hypothesised a similar effect in our system. However, neutralisation of VEGF in AEC-CM indicated that VEGF did not influence the M2 polarising capacity of AEC-CM, which further illustrates the complexity of the AEC secretome (9). Besides M-CSF and VEGF, a range of other factors including IL-10, IL-34, TGF-β and CXCL16 have been shown to induce M2 subtypes during pregnancy (16, 26, 37). Both IL-10 and latency-associated peptide TGF-β were included in our proteomic analysis, but only latency-associated peptide TGF-β could be detected. Hence, future efforts could focus on the M2-inducing capacity of TGF-β. Besides the direct M2-polarising effect of soluble mediators secreted from AECs, we also hypothesised that extracellular vesicles (EVs) could be involved in the observed M2 induction, since EVs from AECs could be involved in immunomodulation (9, 13, 32) and have been suggested for the treatment of inflammatory conditions (38). Fractionation of AEC-CM revealed that isolated EVs did not achieve M2 induction in our *in vitro* assay, while the EV-depleted fraction of AEC-CM efficiently mediated M2 polarisation. This supports the role of AEC-derived M-CSF and other secreted M2-polarising factors. Our findings contrast with one study where AEC-derived exosomes increased the percentage of cells expressing the M2-marker CD206 in murine bone marrow-derived macrophages (11). Still, our findings are partially supported by another study on murine bone-marrow derived cells that demonstrated that co-culture with AEC-CM and AEC-derived EVs as well as AEC-CM depleted of EVs polarised macrophages towards an M2 phenotype (23). These contradictory results may be the consequence of varying AEC culture conditions or differences in experimental procedures, since in our study, human monocytes were treated with AEC-CM right from the beginning of differentiation rather than adding AEC-CM later during the process. Furthermore, the EV cargo is highly dependent on the cell of origin as well as *ex vivo* culture conditions (32, 39), which could explain differences between studies. In summary, our results support that a paracrine effect mediated by human AECs is limited to soluble molecules, suggesting a local effect on macrophages via mediators secreted independently of EVs.

Due to their localisation, AECs could exert an M2-polarising effect on macrophages during embryonic development, given that Langerhans cells, microglia, cardiac macrophages as well as many tissue-resident macrophages are derived from yolk sac or foetal macrophages (40). AECs differentiate already around day 8 of human pregnancy (8), whereas yolk sac macrophages are generated in the first wave of embryonic haematopoiesis at 2.5 weeks of human pregnancy (41), providing AECs at least in theory with the ability to influence these yolk sac macrophages, for example via M-CSF secreted into amniotic fluid (42, 43). However, it is critical to bear in mind that our study was carried out using human AECs isolated from term placentas, and AEC properties and, more relevantly, the AEC secretome may vary during gestation. Nevertheless, human AECs could have lasting effects on phenotypes of tissue-resident macrophages, and given that tissue-resident macrophages can suppress inflammation and restore tissue homeostasis, the potential role of AECs in macrophage polarisation is intriguing (44). In summary, the immunomodulatory potential of AECs and AEC-secreted mediators warrants further in-depth investigation to pave the way for novel treatment strategies and advanced therapies for inflammatory and autoimmune conditions.

Overall, our findings and findings from others highlight the importance of optimisation and standardisation of culture conditions for AECs for future studies on cultured AECs, but also point to the pivotal role of AECs in modulation of the maternal and foetal immunity at the foetal-maternal interface, as well as their potential application in advanced immunomodulatory treatments (7, 8).

There are a few limitations to this study. For the initial comparison of AEC culture conditions, namely normoxic *versus* hypoxic cultivation and 3 *versus* 7 days of culture, a rather low number of samples was used. However, all samples were paired, thus collected from cells isolated and cultured under the same conditions for every donor, which allows statistically valid conclusions on the culture conditions optimally inducing M2-macrophage polarisation. Furthermore, the use of AEC-CM from normoxic culture could be considered a limitation of our study, since hypoxic cell culture most closely resembles the physiological state *in vivo* (45). However, in our *in vitro* model and in protein profiling, we did not observe any difference between AEC-CM obtained from normoxic compared to hypoxic culture. Regarding the inability of EVs to induce M2 polarisation, our results do not exclude the possibility that higher EV concentrations could indeed affect macrophage phenotypes. This remains to be addressed in future studies.

Future perspectives include pinpointing the exact M2-inducing mediators in the EV-depleted fraction of AEC-CM. Furthermore, it could be interesting to investigate whether the EV-enriched fraction just fails to inhibit GM-CSF-induced M1-polarisation, as observed in this study, or if it could even achieve to overcome M-CSF-induced M2-polarisation; hence pointing to differing immunomodulatory effects of the EV-enriched and -depleted fraction of AEC-CM. Furthermore, we limited our investigation to the paracrine effects of AECs by using AEC-CM and AEC-derived EVs, but it would be interesting to investigate the direct cellular actions of AECs on immune and other stromal cells.

## Data Availability

The authors acknowledge that the data presented in this study must be deposited and made publicly available in an acceptable repository, prior to publication. Frontiers cannot accept a manuscript that does not adhere to our open data policies.
