# Supplementary material for: Human amnion epithelial cells induce M2 macrophage polarisation partially via M-CSF secretion but independently of extracellular vesicles in vitro
Source: Front Immunol. 2026 Jan 29;17:1723968. doi: 10.3389/fimmu.2026.1723968 (PMC12894031; doi:10.3389/fimmu.2026.1723968)
Supplement: Supplementary file 1 [file DataSheet1.pdf]

Supplementary Material

Supplementary Material

1 Supplementary Figures and Tables

1.1 Supplementary Figures

Supplementary Figure S1

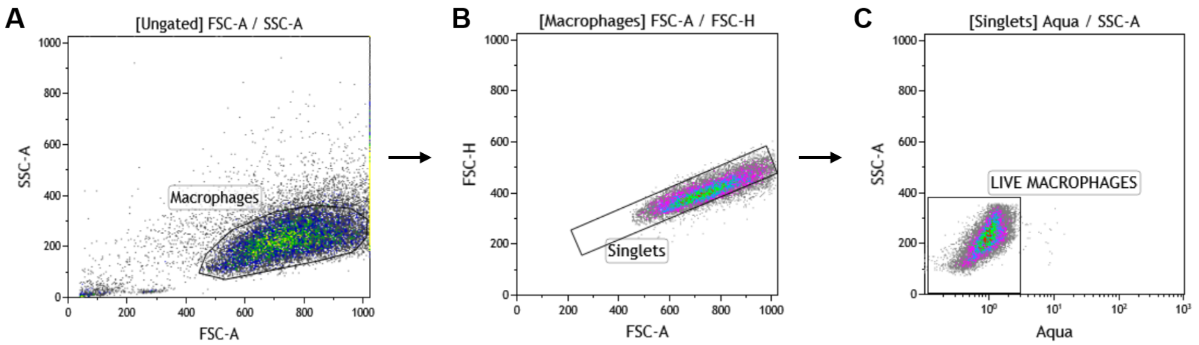

Supplementary Figure S1. Gating strategy for macrophages. *Human CD14$^+$ monocytes were isolated from PBMCs and cultured in the presence of GM-CSF and with or without different concentrations of AEC-CM. After 6 days, macrophages were harvested and stained for flow cytometry. The gating strategy for singlet, live macrophages is shown for one representative sample, where the macrophage population was selected based on forward scatter (FSC-A) and side scatter (SSC-A), as shown in A. This population was then gated for singlets based on FSC-A and FSC-H (B), and finally for live cells only, based on absence of staining with the Aqua Live/Dead Cell Stain (C). This population ("live macrophages") was then analysed for the expression of phenotypic macrophage markers, as shown in Figure 1A.*

Supplementary Figure S2

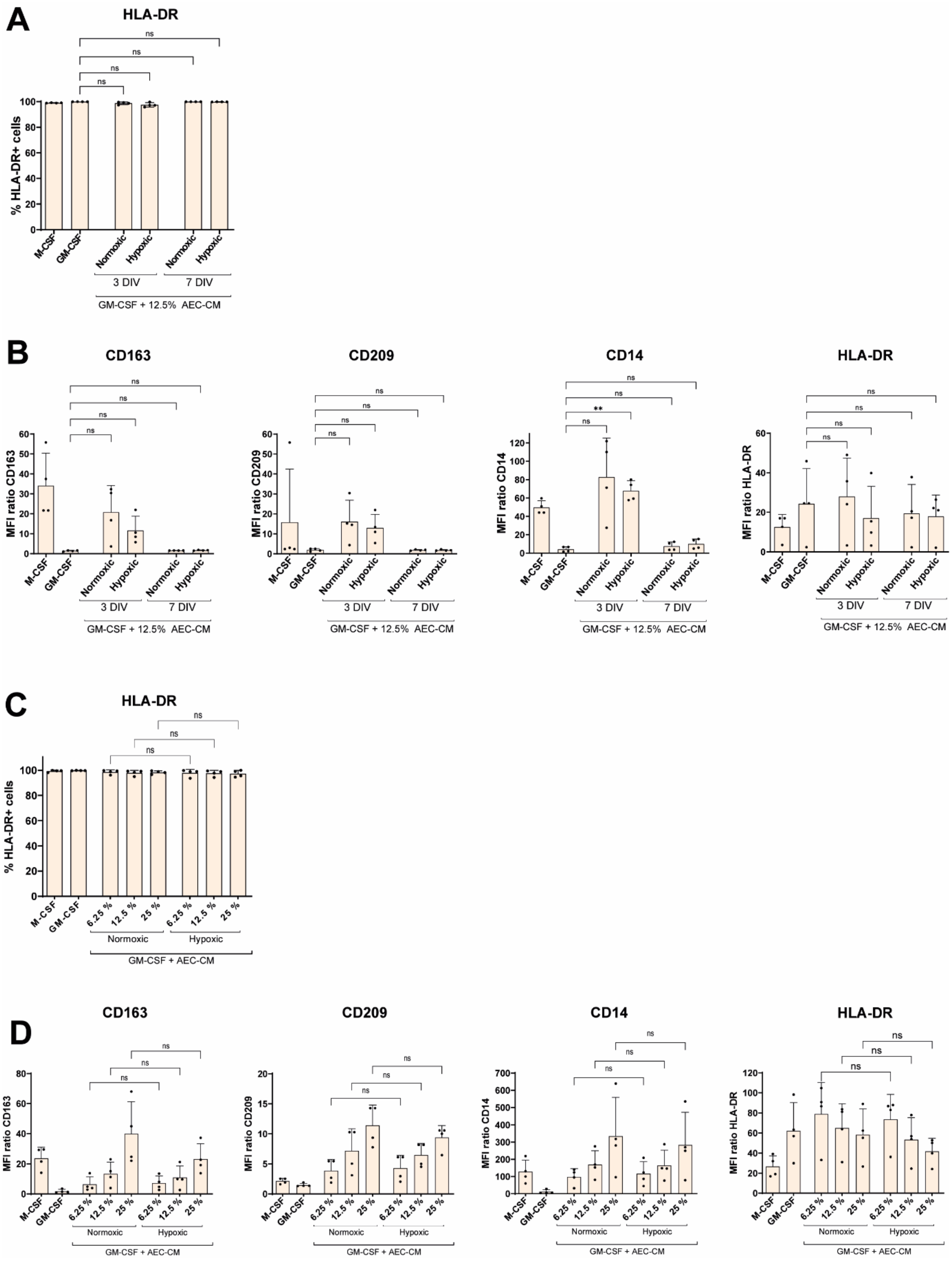

Supplementary Figure S2. *Proportion of HLA-DR expressing cells and MFI ratio for the cell surface markers CD163, CD209, CD14 and HLA-DR from the macrophage polarisation assay with AEC-CM in different culture conditions.* ***A)*** *AEC-CM from normoxic and hypoxic culture were compared in terms of % of HLA-DR-positive cells.* ***B)*** *Median fluorescent intensity (MFI) of CD163, CD209, CD14 and HLA-DR from the same experiments as in Figure 1B and Supplementary Figure S2A.* ***C)*** *Proportion of cells positive for HLA-DR when 6.25%, 12.5% and 25% AEC-CM from 3 days of normoxic and hypoxic culture were added to monocytes.* ***D)*** *MFI of the markers CD163, CD209, CD14 and HLA-DR from the same experiments as in Figure 1C and Supplementary Figure S2C. Statistical differences between samples treated with GM-CSF and AEC-CM and samples treated with GM-CSF only were evaluated using repeated measures ANOVA with Dunnett's multiple comparison test. Differences between samples treated with AEC-CM from normoxic and hypoxic culture were tested with repeated measures ANOVA with Sidak's multiple comparison test. All bars and error bars represent mean and standard deviation. M-CSF was included as a positive control for M2-induction and was not included in the statistical evaluation. ns = non-significant, ** = p<0.01.*

Supplementary Figure S3

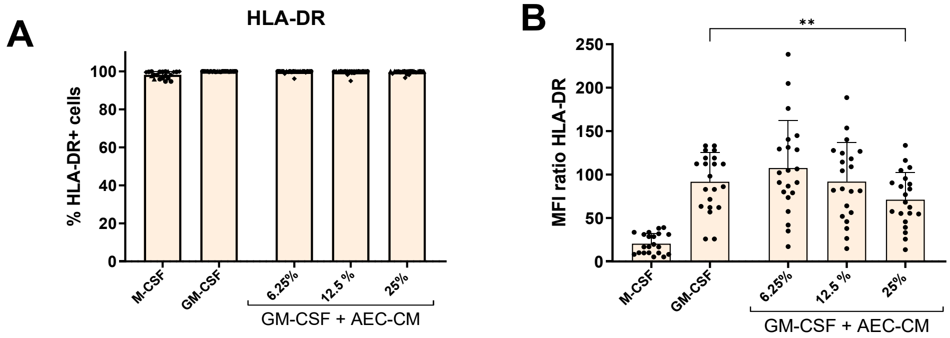

Supplementary Figure S3. *AEC-CM at concentrations of 6.25%, 12.5% and 25% from 3 days of normoxic culture was added to monocytes treated with GM-CSF to test if AEC-CM could override the effect of the M1-inducing growth factor GM-CSF.* ***A)*** *Proportion of positive cells gated for the marker HLA-DR.* ***B)*** *The cellular expression measured as MFI of HLA-DR. Statistical differences were tested using repeated measures ANOVA with Dunnett's multiple comparison test. ** p<0.01. M-CSF was used as a control for M2-induction and was not included in the statistical evaluation.*

Supplementary Figure S4

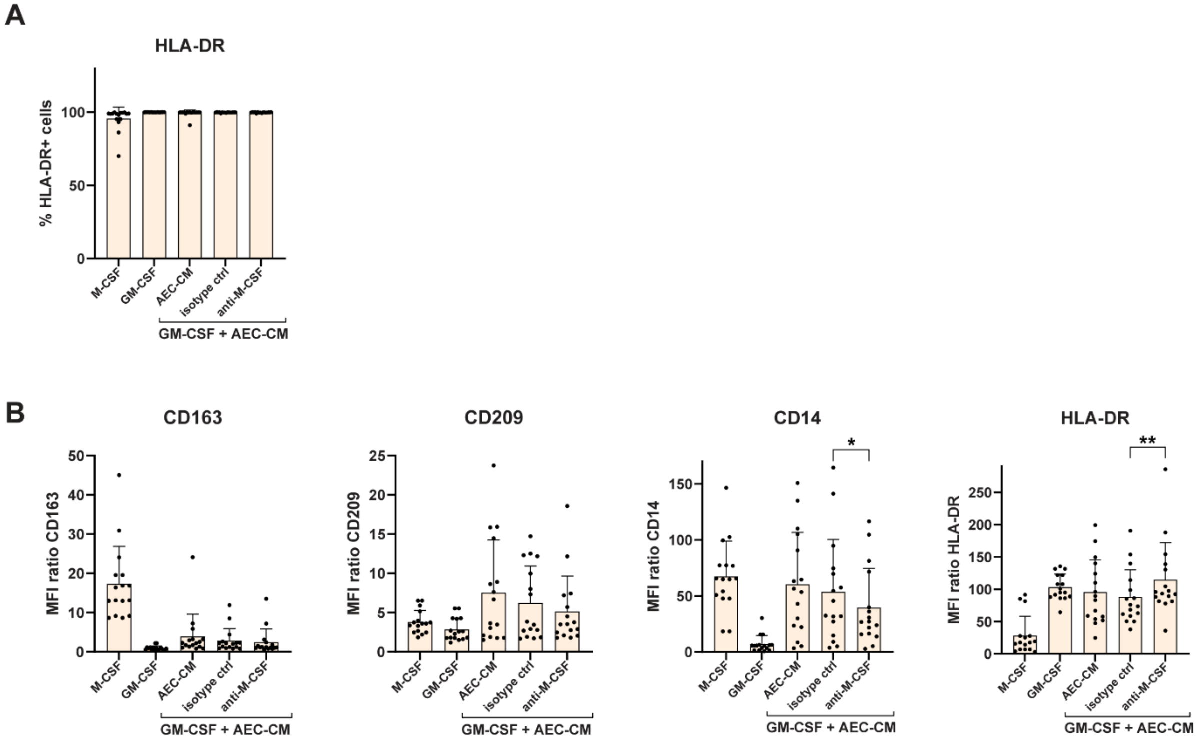

Supplementary Figure S4. *Effects of M-CSF-blocking in AEC-CM on proportion of HLA-DR-positive cells (**A**) and MFI ratio of CD163, CD209, CD14 and HLA-DR (**B**). AEC-CM (at 12.5 or 25%) from 3 days of normoxic culture was added to blood monocytes cultured with M1-inducing GM-CSF in the presence or absence of anti-M-CSF antibody or a corresponding isotype control antibody. Statistical differences were tested using a paired sample t test comparing AEC-CM with anti-M-CSF antibody and its corresponding isotype control antibody. Bars and error bars represent mean and standard deviation. * p<0.05, ** p<0.01.*

Supplementary Figure S5

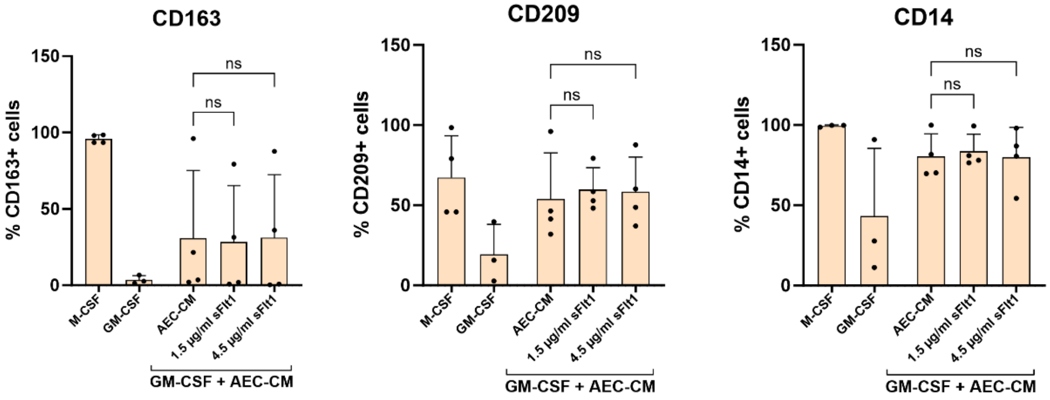

Supplementary Figure S5. *Blocking of VEGF in macrophage polarisation. Monocytes were polarised using the M1-inducer GM-CSF and treated with 25% AEC-CM (from 3 days of AEC culture in normoxic conditions) and 1.5 or 4.5 µg/ml of the soluble VEGF receptor sFlt1. An M-CSF treated sample was added as a control for M2 induction. The proportion of cells positive for CD163, CD209 and CD14 are shown. Statistical differences were tested using repeated measures ANOVA with Dunnett's multiple comparison test. Bars and error bars indicate means and standard deviations. n=4, ns = non-significant.*

Supplementary Figure S6

A

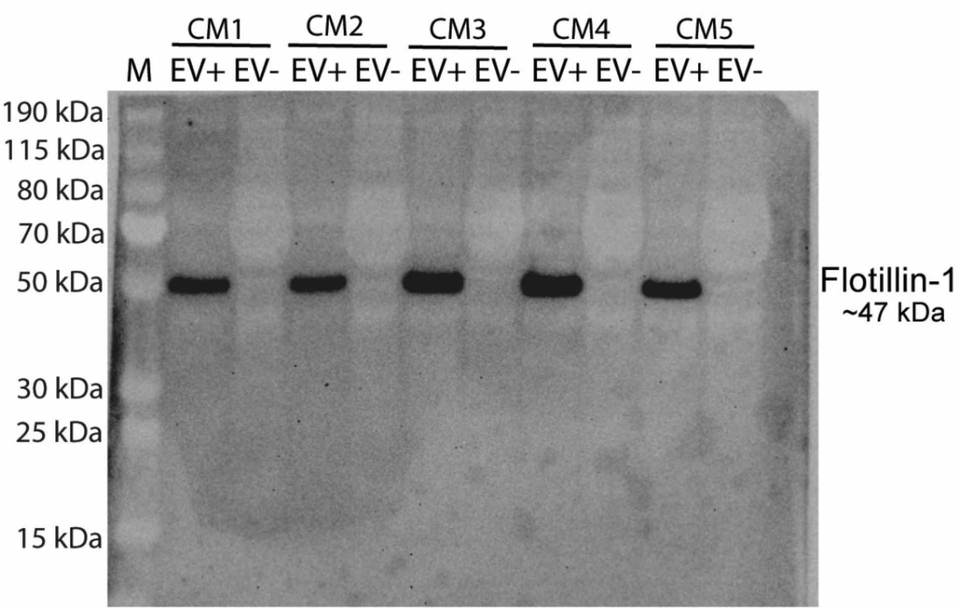

B

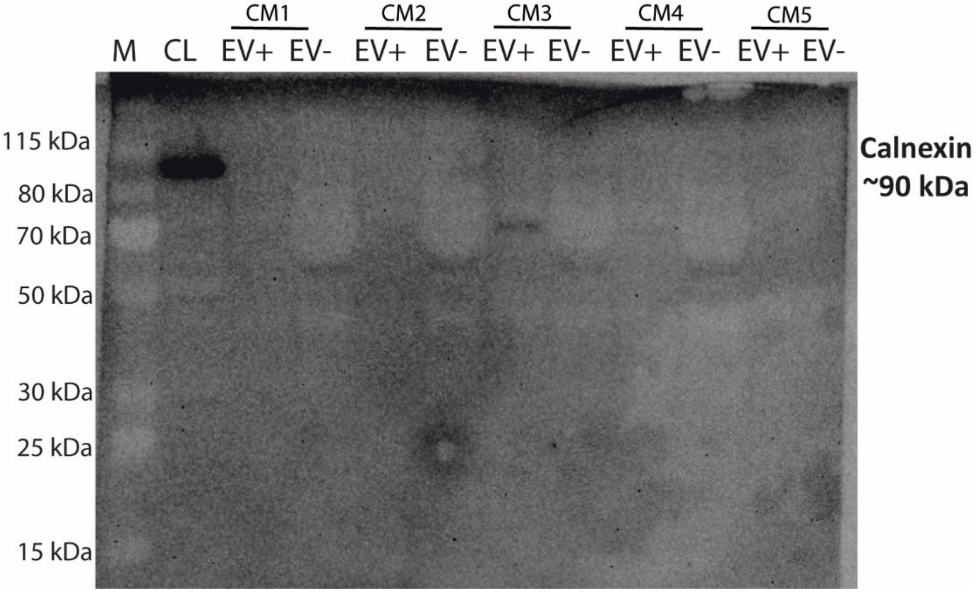

Supplementary Figure S6. *Western blots showing the EV marker flotillin-1 (**A**, cropped blot in Figure 4A) and the negative control calnexin (**B**, marker for cell contamination) in the EV-enriched (EV+) and the EV-depleted (EV-) fraction of 5 different randomly selected AEC samples. CM = conditioned medium from AE cells. CL = cell lysate of enteric glial cells.*

Supplementary Figure S7

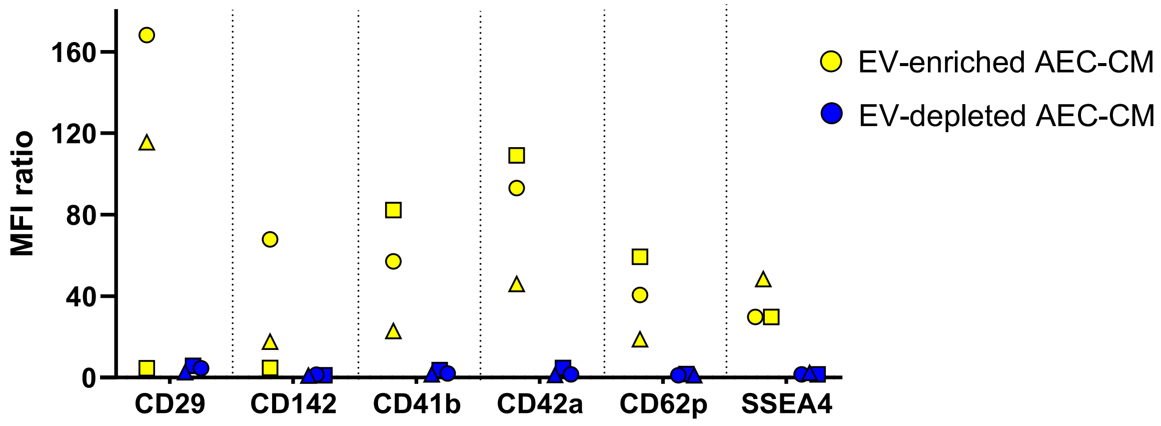

Supplementary Figure 7. *Flow cytometry for EV-related markers was performed on the EV-enriched and EV-depleted fraction of three AEC-CM samples after isolation of EVs. The AEC-CM samples were randomly selected. Each symbol represents one individual sample.*

Supplementary Figure S8

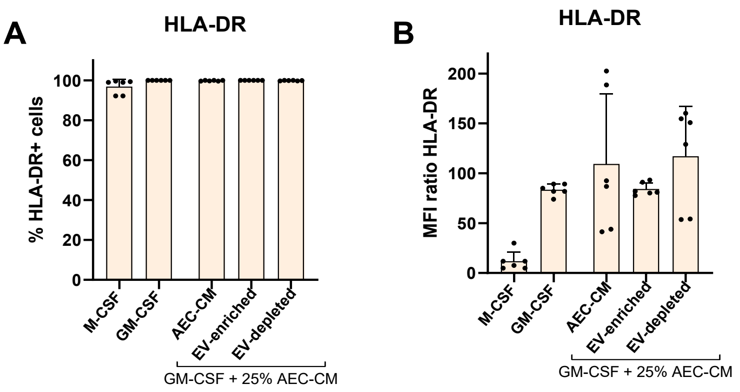

Supplementary Figure 8. *EV-enriched and EV-depleted fractions were isolated from AEC-CM to compare M2-inducing capacity.* ***A)*** *Percentage of cells positive for HLA-DR.* ***B)*** *MFI ratio for HLA-DR. Statistical difference was tested using repeated measures ANOVA with Dunnett's multiple comparison test. Bars and error bars represent mean and standard deviation. N=6. AEC-CM refers to the original sample of conditioned medium.*

1.2 Supplementary Tables

Supplementary Table 1

Supplementary Table 1. *Protein levels from the proximity extension assay (Olink Target 96 Inflammation panel) performed on conditioned medium from amnion epithelial cells cultured for three (3DIV) or seven (7DIV) days in normoxic or hypoxic conditions (n=4 for each condition). Protein levels are expressed as NPX values on a log2 scale. To calculate ΔNPX values for normoxic and hypoxic culture conditions shown in Figure 1E, the NPX values of the 7DIV sample was subtracted from the 3DIV sample from each donor. A median ΔNPX value was then calculated for each condition. In Figure 1E, proteins are sorted based on the median of ΔNPX values. Red colour depicts NPX values below 0 (thus, where the NPX value of the protein is higher in 7DIV than in 3DIV), and green colours depicts NPX values above 0. Proteins are ranked alphabetically and only proteins with less than 50% missing values (n=41) are included.*

Olink protein name	Alternative protein name	Protein name used in this study	Uniprot ID	Normoxic 3DIV	Normoxic 7DIV	ΔNPX normoxic	Hypoxic 3DIV	Hypoxic 7DIV	ΔNPX hypoxic	ΔNPX (total)
**4E-BP1**		**4E-BP1**	Q13541	3.17	6.96	-3.94	2.77	6.69	-4.26	-3.94
**ADA**		**ADA**	P00813	1.12	1.77	-0.73	1.23	1.85	-0.67	-0.67
**Beta-NGF**		**Beta-NGF**	P01138	0.69	0.04	0.7	0.5	0.08	0.54	0.64
**CASP-8**		**CASP-8**	Q14790	0.97	0.87	0.03	0.84	1.3	-0.43	-0.22
**MCP-4**	**CCL13**	**CCL13**	Q99616	5.39	3.14	2.17	5.68	3.7	2.04	2.12
**MCP-1**	**CCL2**	**CCL2**	P13500	4.95	2.05	2.17	5.95	3.1	2.22	2.17
**CCL20**		**CCL20**	P78556	10.65	6.89	3.83	11.56	7.95	3.85	3.83
**CCL23**		**CCL23**	P55773	0.03	-0.56	0.59	0.1	-0.56	0.58	0.58

Olink protein name	Alternative protein name	Protein name used in this study	Uniprot ID	Normoxic 3DIV	Normoxic 7DIV	ΔNPX normoxic	Hypoxic 3DIV	Hypoxic 7DIV	ΔNPX hypoxic	ΔNPX (total)
**CCL28**		**CCL28**	Q9NRJ3	1.03	1.14	-0.04	0.91	0.96	-0.09	-0.07
**CCL4**		**CCL4**	P13236	1.8	-0.42	2.11	2.05	-0.06	1.92	1.93
**CD40**		**CD40**	P25942	9.28	10.54	-1.25	8.87	10.81	-1.97	-1.4
**CDCP1**		**CDCP1**	Q9H5V8	4.12	2.37	1.91	4.06	3.11	1.17	1.72
**CST5**		**CST5**	P28325	2.15	1.24	0.97	1.68	1.08	0.47	0.82
**CXCL1**		**CXCL1**	P09341	12.44	8.35	4.11	12.74	8.4	4.3	4.24
**CXCL11**		**CXCL11**	O14625	1.69	0.34	1.42	2.2	0.57	1.41	1.41
**CXCL5**		**CXCL5**	P42830	4.41	2.86	1.65	4.39	3.07	1.59	1.65
**CXCL6**		**CXCL6**	P80162	2.44	0.53	2.24	2.52	0.81	1.9	1.99
**IL8**	**CXCL8**	**CXCL8**	P10145	12.06	8.18	4.07	12.32	8.48	3.86	3.98
**DNER**		**DNER**	Q8NFT8	-0.14	-0.52	0.43	-0.21	-0.74	0.45	0.45
**FGF-19**		**FGF-19**	O95750	2.96	0.1	2.9	2.59	0.57	2.03	2.48
**Flt3L**		**Flt3L**	P49771	2.02	2.4	-0.27	2.2	2.07	0.15	-0.07
**HGF**		**HGF**	P14210	1.43	0.54	0.9	0.74	0.67	0.41	0.62
**IL-12B**		**IL-12B**	P29460	0.96	-0.38	1.59	0.55	-0.33	1.02	1.29
**IL18**	**IL-18**	**IL-18**	Q14116	0.18	-0.44	0.64	0.25	-0.28	0.36	0.46
**IL-18R1**		**IL-18R1**	Q13478	2.33	1.74	0.54	2.1	2.16	-0.24	0.15
**IL6**	**IL-6**	**IL-6**	P05231	5.94	2.51	3.63	6.62	3.08	3.55	3.63
**LAP TGF-beta-1**		**LAP TGF-beta-1**	P01137	2.58	0.65	1.79	2.45	0.66	1.66	1.69
**LIF**		**LIF**	P15018	3.47	-0.03	3.56	4.07	0.37	3.94	3.75

Olink protein name	Alternative protein name	Protein name used in this study	Uniprot ID	Normoxic 3DIV	Normoxic 7DIV	ΔNPX normoxic	Hypoxic 3DIV	Hypoxic 7DIV	ΔNPX hypoxic	ΔNPX (total)
**CSF-1**	**M-CSF**	**M-CSF**	P09603	6.01	4.76	1.1	5.74	4.25	1.79	1.62
**MMP-1**		**MMP-1**	P03956	7.65	4.74	2.59	7.63	4.53	2.67	2.63
**MMP-10**		**MMP-10**	P09238	7.1	3.3	3.48	7.98	3.66	3.91	3.59
**OPG**		**OPG**	O00300	1.05	-0.11	1.07	1.14	0.11	0.75	1.02
**PD-L1**		**PD-L1**	Q9NZQ7	2.92	1.88	1.15	3.22	2.64	0.73	0.96
**SCF**		**SCF**	P21583	3.68	2.82	1.02	3	2.22	0.79	0.86
**STAMBP**		**STAMBP**	O95630	1.17	2.51	-1.34	0.99	2.6	-1.62	-1.41
**TGF-alpha**		**TGF-alpha**	P01135	1.99	-0.07	2.19	2.12	0.09	2.26	2.19
**TNF**		**TNF**	P01375	1.98	-0.48	2.38	1.8	-0.43	2.44	2.44
**TNFRSF9**		**TNFRSF9**	Q07011	3.21	1.3	1.98	3.48	1.29	2.31	2.06
**TWEAK**		**TWEAK**	O43508	3.39	2.18	1.13	3.39	3.39	0.12	0.54
**uPA**		**uPA**	P00749	12.24	9.6	2.09	11.08	9.54	2.2	2.11
**VEGFA**		**VEGFA**	P15692	12.7	10.64	2.08	13.36	11.79	1.49	1.9

Supplementary Table 2

Supplementary Table 2. *Protein levels from the proximity extension assay (Olink Target 96 Inflammation panel) performed on conditioned medium from amnion epithelial cells cultured for 3 days in normoxic conditions (n=22). Protein levels are expressed as NPX values on a log2 scale. Mean NPX values for all proteins with less than 50% missing values (n=59) are shown. Proteins are ranked by mean NPX in descending order.*

Olink protein name	Alternative protein name	Protein name used in this manuscript	Uniprot ID	NPX (mean)
uPA		uPA	P00749	12.36
MMP-1		MMP-1	P03956	12.29
VEGFA		VEGFA	P15692	12.14
CD40		CD40	P25942	11.42
IL8	CXCL8	CXCL8	P10145	10.02
CXCL1		CXCL1	P09341	9.68
MCP-4	CCL13	CCL13	Q99616	9.4
CXCL5		CXCL5	P42830	8.82
MCP-1	CCL2	CCL2	P13500	7.73
CSF-1	M-CSF	M-CSF	P09603	6.43
TWEAK		TWEAK	O43508	6.13
MMP-10		MMP-10	P09238	6.06
CCL20		CCL20	P78556	5.83
CDCP1		CDCP1	Q9H5V8	5.59
CCL23		CCL23	P55773	5.43
IL6	IL-6	IL-6	P05231	5.31
LAP TGF-beta-1		LAP TGF-beta-1	P01137	5.2
HGF		HGF	P14210	5.03
Flt3L		Flt3L	P49771	5.03
CASP-8		CASP-8	Q14790	4.89
CCL3		CCL3	P10147	4.79
CXCL6		CXCL6	P80162	4.68
IL18	IL-18	IL-18	Q14116	4.64

Olink protein name	Alternative protein name	Protein name used in this manuscript	Uniprot ID	NPX (mean)
OPG		OPG	O00300	4.61
FGF-19		FGF-19	O95750	4.55
STAMBP		STAMBP	O95630	4.48
TGF-alpha		TGF-alpha	P01135	4.25
ADA		ADA	P00813	4.21
SCF		SCF	P21583	4.1
DNER		DNER	Q8NFT8	3.9
4E-BP1		4E-BP1	Q13541	3.88
ST1A1		ST1A1	P50225	3.59
CST5		CST5	P28325	3.55
MCP-2	CCL8	CCL8	P80075	3.42
AXIN1		AXIN1	O15169	3.23
TRAIL		TRAIL	P50591	3.17
CCL4		CCL4	P13236	3.15
PD-L1		PD-L1	Q9NZQ7	3
IL-18R1		IL-18R1	Q13478	2.97
CXCL11		CXCL11	O14625	2.86
CXCL9		CXCL9	Q07325	2.86
CXCL10		CXCL10	P02778	2.85
IL-12B		IL-12B	P29460	2.75
CCL19		CCL19	Q99731	2.7
SIRT2		SIRT2	Q8IXJ6	2.67
IL-10RB		IL-10RB	Q08334	2.53
LIF		LIF	P15018	2.5
TNFSF14		TNFSF14	O43557	2.49
CD8A		CD8A	P01732	2.46
CD244		CD244	Q9BZW8	2.31
CCL11		CCL11	P51671	2.06
TNFRSF9		TNFRSF9	Q07011	1.9
CD5		CD5	P06127	1.78
TNF		TNF	P01375	1.6
EN-RAGE		EN-RAGE	P80511	1.41

Olink protein name	Alternative protein name	Protein name used in this manuscript	Uniprot ID	NPX (mean)
OSM		OSM	P13725	1.37
IL7	IL-7	IL-7	P13232	1.12
CCL28		CCL28	Q9NRJ3	1.07
CD6		CD6	P30203	0.96
